# Correlation Between Shoulder and Wrist Muscle Flexibility, Hand Muscle Strength, and Smash Accuracy in Sub-elite Badminton Players

**DOI:** 10.7759/cureus.115083

**Published:** 2026-08-24

**Authors:** Shantanu Kshirsagar, Smita C Patil

**Affiliations:** 1 Department of Sports Physiotherapy, Krishna College of Physiotherapy, Krishna Vishwa Vidyapeeth (Deemed to Be University), Karad, IND

**Keywords:** badminton, grip strength, shoulder flexibility, smash accuracy, wrist flexibility

## Abstract

Background

Badminton is an intense racket sport that requires a variety of physical fitness, technical proficiency, and neuromuscular coordination. The smash is regarded as the most powerful offensive stroke in badminton, and its effectiveness depends on several physical factors, including upper-extremity flexibility and strength. Although shoulder and wrist flexibility, as well as hand muscle strength, are believed to influence stroke performance, there is little information available about their relationship with smash accuracy in sub-elite badminton players.

Methods

A cross-sectional correlational study was carried out among 28 sub-elite badminton players aged 18-24 years recruited from badminton academies and sports training centers in Karad, Maharashtra, India. Convenience sampling was used to choose participants based on predefined inclusion and exclusion criteria. Shoulder and wrist muscle flexibility were evaluated using the Prone Stick Test, hand muscle strength was assessed with the Maximum Push-Up Test, and smash accuracy was measured using a standardized Smash Accuracy Test. Pearson’s correlation coefficient was utilized to analyze the association between variables, with the degree of importance set at p < 0.05.

Results

The average age of participants was 21.07 ± 1.82 years, with a mean playing experience of 3.96 ± 1.47 years. A somewhat favorable association was discovered between shoulder flexibility and smash accuracy (r=0.58, p=0.001). Wrist flexibility demonstrated a fair positive correlation with smash accuracy (r = 0.41, p = 0.030). Hand muscle strength showed a strong positive correlation with smash accuracy (r=0.69, p<0.001), demonstrating that individuals with higher upper-extremity strength tended to demonstrate better smash accuracy. Among the variables studied, hand muscle strength exhibited the strongest association with smash accuracy.

Conclusion

Shoulder flexibility, wrist flexibility, and hand muscle power were substantially correlated with smash accuracy in sub-elite badminton players. Hand muscle strength demonstrated the strongest relationship with smash accuracy, followed by shoulder flexibility and wrist flexibility. These findings suggest that upper-extremity flexibility and strength are significant physical characteristics connected to badminton smash performance. Incorporating flexibility and strengthening exercises into training programs may help improve smash accuracy and overall playing performance in sub-elite badminton players.

## Introduction

Badminton is among the most popular racket sports worldwide and demands a blend of technical proficiency, tactical awareness, physical fitness, and psychological preparedness. The sport is characterized by rapid multidirectional movements, repeated accelerations and decelerations, jumping, lunging, and overhead strokes performed at high speed. Successful performance in badminton depends on the effective integration of physiological, biomechanical, and neuromuscular factors that allow athletes to execute sport-specific skills with precision and consistency. The scientific understanding of badminton has highlighted the importance of physical and biomechanical characteristics in determining competitive performance [1].

Among the various strokes used in badminton, the smash is considered the most powerful offensive technique and is frequently utilized to gain an advantage over competitors during rallies. The efficiency of a smash depends not only on the ability to create force and racket-head velocity but also on the accuracy with which the shuttlecock is directed toward targeted areas of the opponent’s court. Previous research has demonstrated a significant relationship between upper-extremity muscle performance and racket velocity during badminton smash movements, suggesting that physical characteristics of the upper limb contribute to stroke execution [2].

The repetitive overhead nature of badminton places substantial demands on the shoulder complex. Consequently, shoulder-related musculoskeletal problems are commonly reported among badminton players. Research on professional athletes has documented a high prevalence of shoulder pain associated with repetitive overhead activity, emphasizing the importance of maintaining optimal shoulder function for both performance enhancement and injury prevention [3]. Further evidence has identified shoulder mobility and functional limitations as possible hazards for shoulder pain in young badminton players, highlighting the need to assess upper-extremity physical characteristics in this population [4].

Competitive badminton requires players to repeatedly perform explosive movements and technically demanding strokes throughout prolonged match durations. Analysis of elite-level competition has shown that athletes must maintain stroke quality and precision despite increasing physical demands and fatigue [5]. Reviews of racket sports have consistently emphasized the contribution of strength, flexibility, and neuromuscular control to successful performance, particularly during overhead actions requiring high levels of coordination and force production [6]. Biomechanical investigations of the badminton overhead smash have additionally shown that efficient stroke execution relies on coordinated movement throughout the kinetic chain, enabling efficient transfer of energy from proximal to distal segments and resulting in greater racket-head velocity and shuttlecock control [7].

Shoulder flexibility is considered an important component of overhead athletic performance because it allows athletes to achieve optimal joint positioning during movement. Adequate mobility contributes to efficient biomechanics and may enhance force production during sport-specific activities. Evidence from shoulder rehabilitation research supports the importance of maintaining flexibility to optimize upper-extremity function [8]. Repetitive participation in overhead sports may result in alterations in shoulder range of motion, including deficits in glenohumeral internal rotation and total rotational movement [9]. Additionally, tightness in the posterior shoulder has been associated with altered shoulder mechanics and may negatively influence upper-extremity performance [10]. Comparisons between athletic and non-athletic populations further suggest that sport-specific adaptations influence shoulder mobility and flexibility characteristics [11].

In addition to flexibility, hand muscle strength may be crucial in badminton performance. Hand-grip dynamometry is widely accepted as a reliable measure of upper-extremity strength and muscular function [12]. Studies have shown that grip strength is positively associated with overall muscular strength in healthy individuals, indicating its value as an indicator of functional upper-limb performance [13]. Furthermore, hand-grip strength has been linked to various athletic performance outcomes, including force production, power generation, and sport-specific skill execution, suggesting that stronger athletes may have enhanced racket control during badminton play [14].

Flexibility training is commonly incorporated into athletic conditioning programs because of its potential to improve mobility and movement efficiency. Evidence indicates that stretching interventions can influence physical performance through improvements in range of motion and neuromuscular function [15]. Systematic reviews have also emphasized the relationship between flexibility and athletic performance, emphasizing the importance of maintaining adequate mobility for efficient movement patterns [16]. Research has additionally shown that increased range of motion may help with enhanced performance and reduced injury risk among athletes [17]. Studies investigating the mechanisms of flexibility improvements have reported significant increases in range of motion of the joint following stretching interventions, reinforcing the significance of flexibility assessment in sports performance research [18].

Upper-body strength has been found to have an impact on sport-specific performance variables in racket sports. Research involving tennis players reported positive relationships between upper-body strength, power, and serve velocity, indicating that muscular strength contributes significantly to overhead stroke performance [19]. Similarly, performance analysis studies in badminton have identified stroke effectiveness as a key determinant of competitive success, emphasizing the importance of executing accurate and powerful shots during match play [20].

Despite the recognized importance of flexibility and strength in overhead sports, there is scant information on the combined relationship between shoulder flexibility, wrist flexibility, hand muscle strength, and smash accuracy in sub-elite badminton players. Understanding these associations may assist sports physiotherapists, coaches, and athletes in developing targeted training strategies to optimize performance and reduce injury risk. Therefore, the present research seeks to ascertain the correlation between shoulder and wrist muscle flexibility, hand muscle strength, and smash accuracy in sub-elite badminton competitors.

## Materials and methods

Study design and study setting

This cross-sectional correlational analysis was carried out to determine the link between shoulder muscle flexibility, wrist muscle flexibility, hand muscle strength, and smash accuracy among sub-elite badminton players. The investigation was conducted at Chhatrapati Shivaji Stadium and affiliated badminton academies in Karad, Maharashtra, India. Data gathering took place over a period of one year.

Convenience sampling was used to find participants based on predefined inclusion and exclusion criteria. Eligible players underwent assessment of shoulder flexibility, wrist flexibility, hand muscle strength, and smash accuracy. The gathered information was examined to determine the correlation between the physical variables and smash accuracy.

Participants

The research comprised 28 sub-elite badminton players between the ages of 18 and 24. Sub-elite badminton players were described as individuals who regularly participated in district-, university-, state-, or club-level badminton competitions but were not classified as elite or international-level athletes. Participants were recruited from Chhatrapati Shivaji Stadium and affiliated badminton academies in Karad, using a convenience sampling method.

All participants who met inclusion as well as exclusion standards were informed about the study procedures and gave written, informed consent before enrollment. Demographic data, such as body mass index, age, sex, and playing experience, were recorded before assessment.

The sample size was determined by utilizing the formula $\mathrm{n} = \left( \frac{\mathrm{C} \times \mathrm{Z}_{\alpha/2} + \mathrm{Z}_{\beta}}{3} \right)^{2}$

where n represents the required sample size, C represents the constant/coefficient used in the sample size calculation, $Z_{\alpha/2}$ represents the standard normal deviate corresponding to the selected two-sided significance level *α*. For a significance level of 5%, *α = 0.05 *, the value is 1.96. *α* represents the probability of a Type I error and was set at 0.05. $Z_{\beta}$ represents the standard normal deviate corresponding to the desired statistical power. For a power of 80%, *β = 0.20*, the value is 0.84. *β* represents the probability of a Type II error. 3 represents the study-specific parameter used in the denominator of the formula.

Inclusion criteria

Participants who qualified for inclusion in the study were sub-elite badminton players aged between 18 and 24 years who regularly participated in training and competitive badminton but had not competed at the international level. Players were required to have a minimum of one year of consistent badminton training experience and engage in regular training for at least eight hours per week. Only those who regularly performed smash strokes during training and competition and were willing to undergo shoulder and wrist flexibility assessments as well as hand muscle strength testing were included. Both left-handed and right-handed players were thought to be qualified for participation. Furthermore, each participant had to provide informed consent and express their willingness to take part in the research.

Exclusion criteria

Participants were not included in the research if they had sustained any recent injury affecting the shoulder, wrist, or hand within the previous six months. People having a history of musculoskeletal or neurological conditions that may affect upper-extremity function, flexibility, strength, or sports performance were also excluded. Players who were currently undergoing any form of medical treatment or rehabilitation for sports-related or musculoskeletal conditions were deemed ineligible for participation. Additionally, individuals presenting with congenital deformities of the spine, upper extremity, or lower extremity that could affect movement patterns or performance during assessment were not included in the research.

Outcome measures

Smash Accuracy Test

Smash accuracy was assessed using a standardized badminton smash accuracy test. Participants were instructed to perform smashes toward designated target areas on the court, and scores were awarded based on the accuracy of shuttle placement. The badminton smash accuracy test has demonstrated excellent validity (r = 0.84) and acceptable reliability (r = 0.78) for evaluating smash performance in badminton players [21].

Push-Up Test

The strength of the hand muscles was measured using the Push-Up Test, which is a simple and practical measure of upper-extremity muscular strength and endurance. The Push-Up Test has demonstrated good test-retest reliability (intraclass correlation coefficient (ICC) = 0.93) and is widely used in athletic populations to evaluate upper-body functional strength [22]. Shoulder and wrist muscle flexibility were assessed using standardized flexibility assessment procedures to ascertain the range of motion and flexibility of the upper-extremity musculature. These tests have shown positive dependability in assessing joint mobility, with ICC that was reported in values ranging from 0.80 to 0.95 [23].

Procedure

The research procedure was approved by the Institutional Protocol Committee, and ethical approval was obtained from the Institutional Ethics Committee of Krishna Vishwa Vidyapeeth (Deemed to Be University), Karad, which issued approval 317/2025-2026 before the commencement of the study. Participants were recruited according to the predefined inclusion and exclusion criteria. The study objectives and procedures were explained, and written informed consent was obtained from all participants. Demographic information, including age, sex, body mass index, dominant playing hand, playing experience, and weekly training hours, was recorded.

Before testing, participants performed a standardized five- to 10-minute warm-up consisting of light aerobic activity and upper-limb dynamic stretching. All assessments were conducted in a single session under standardized environmental conditions by the same investigator. Shoulder and wrist flexibility were assessed using the Prone Stick Test. Participants were positioned prone on a treatment table and instructed to hold a standardized stick with both hands while maintaining full elbow extension. They were asked to raise the stick as high as possible without compensatory trunk movement. The dominant upper limb was evaluated, and three trials were performed with a 30-second rest interval between trials. The best performance was recorded for analysis. Hand muscle strength was then assessed using the maximum Push-Up Test, followed by smash accuracy assessment using a standardized smash accuracy test. Adequate rest was provided between assessments to minimize fatigue, and all measurements were documented on a structured data collection form.

Statistical analysis

Statistical analysis was performed using IBM SPSS Statistics for Windows, Version 25.0 (IBM Corp., Armonk, NY, USA). Descriptive statistics were calculated for all study variables. Continuous variables were expressed as mean ± standard deviation (SD), while categorical variables were presented as frequencies and percentages.

Pearson's correlation coefficient (r) was used to determine the relationship between shoulder muscle flexibility, wrist muscle flexibility, hand muscle strength, and smash accuracy. The corresponding correlation coefficient (r), test statistic (t), degrees of freedom (df = n − 2), and p-value were calculated and reported for each correlation analysis in the relevant tables.

All statistical tests were two-tailed, and a p-value of less than 0.01 (p < 0.01) was considered statistically significant.

## Results

The study included 28 sub-elite badminton players. The mean age of the participants was 21.07 ± 1.82 years. The mean body mass index was 22.14 ± 2.08 kg/m². Participants had a mean playing experience of 3.96 ± 1.47 years and trained for an average of 9.18 ± 1.36 hours per week. The descriptive characteristics of the participants are presented in Table 1.

**Table 1:** Participant characteristics (N = 28)

Variable	Mean ± SD
Age (years)	21.07 ± 1.82
Body mass index (kg/m²)	22.14 ± 2.08
Playing experience (years)	3.96 ± 1.47
Training Hours/Week	9.18 ± 1.36

Pearson's correlation analysis demonstrated significant positive relationships between the assessed physical variables and smash accuracy. Shoulder flexibility showed a moderate positive correlation with smash accuracy (r = 0.58, t = 3.64, p = 0.001). Wrist flexibility demonstrated a fair positive correlation (r = 0.41, t = 2.29, p = 0.030). Hand muscle strength measured using the Push-Up Test showed a strong positive correlation with smash accuracy (r = 0.69, t = 4.86, p < 0.001), indicating that greater hand muscle strength was associated with higher smash accuracy. These findings are summarized in Table 2.

**Table 2:** Correlation between physical variables and smash accuracy

Variable	r-value	p-value
Shoulder flexibility	0.58	0.001
Wrist flexibility	0.41	0.030
Hand muscle strength (Push-Up Test)	0.69	<0.001

Based on the strength of the correlation coefficients, shoulder flexibility demonstrated a moderate positive correlation with smash accuracy, wrist flexibility showed a fair positive correlation, and hand muscle strength demonstrated a strong positive correlation. All three variables were significantly associated with smash accuracy, with hand muscle strength showing the strongest relationship, as presented in Table 3.

**Table 3:** Summary of correlation strength

Variable	Correlation Strength	Interpretation
Shoulder flexibility	Moderate positive	Significant
Wrist flexibility	Fair positive	Significant
Hand muscle strength	Strong positive	Highly significant

## Discussion

The present study found a moderately favorable relationship between shoulder flexibility and smash accuracy (r=0.58, p=0.001). Badminton smashes require a large range of motion at the shoulder joint to achieve optimal racket positioning and efficient energy transfer through the kinetic chain. Previous biomechanical studies have demonstrated that overhead smash performance depends on coordinated shoulder movement and adequate joint mobility [7]. Adequate shoulder flexibility allows players to achieve a greater backswing and follow-through, facilitating better shuttle placement and control during smash execution. Deficits in shoulder mobility have been linked to changed movement patterns and reduced upper-extremity performance in overhead athletes [9].

Wrist flexibility demonstrated a fair positive correlation with smash accuracy (r = 0.41, p = 0.030). The wrist contributes significantly to the final acceleration phase of the badminton smash; it is crucial to controlling shuttle direction and racket orientation. Kwan et al. reported that wrist action contributes substantially to shuttlecock velocity generation and stroke control during badminton performance [24]. Furthermore, biomechanical analysis of badminton stroke execution has shown that distal segment motion, particularly wrist movement, contributes to successful smash performance and shot precision [25].

Hand muscle strength showed the strongest positive correlation with smash accuracy (r = 0.69, p < 0.001). This finding suggests that athletes with greater upper-extremity strength are able to generate better racket control and stability during stroke execution. Prior research has shown a positive association between upper-body strength and performance outcomes in racket sports [19]. Awatani et al. reported that shoulder muscle strength is significantly associated with racket velocity during badminton forehand smash movements [2]. Greater muscular strength may enhance force transmission and improve control at the point of shuttle contact, thereby contributing to better smash accuracy. Physical fitness characteristics have demonstrated a significant influence on badminton performance and skill execution [26]. The current study's conclusions are further supported by evidence suggesting that upper-extremity strength and coordination are important determinants of performance in overhead sports [27].

Clinical implications

The results of this investigation suggest that sports physiotherapists, strength and conditioning professionals, and badminton coaches should incorporate upper-extremity flexibility and strengthening exercises into training programs. Regular assessment of shoulder flexibility, wrist flexibility, and hand muscle strength may help identify performance limitations and guide individualized intervention strategies aimed at improving smash accuracy and overall playing performance.

Strengths

A major strength of this investigation was simultaneous evaluation of shoulder flexibility, wrist flexibility, and hand muscle strength in relation to a sport-specific performance outcome. The use of practical and clinically applicable assessment tools increases the relevance of the findings for coaches, physiotherapists, and athletes. Additionally, the study focused on sub-elite badminton players, a population that has received comparatively limited attention in the literature.

Limitations

Several limitations should be taken into account when evaluating the results. The size of the sample was rather small, which could restrict the applicability of the results. The cross-sectional design allows identification of associations but does not establish causality. Furthermore, technical proficiency, reaction time, psychological factors, and biomechanical variables that may influence smash accuracy were not assessed. The study was also limited to sub-elite badminton players from a specific geographical region.

Future scope

Future studies ought to incorporate more sample sizes and players from different competitive levels to improve the generalizability of findings. Longitudinal and interventional studies are needed to determine whether improvements in flexibility and strength directly enhance smash accuracy. Further investigations should also explore the contribution of additional factors such as shoulder strength, core stability, movement coordination, reaction time, and biomechanical characteristics to develop a more thorough comprehension of badminton performance.

## Conclusions

The present research found significant positive correlations between shoulder flexibility, wrist flexibility, hand muscle strength, and smash accuracy among sub-elite badminton players. Hand muscle strength demonstrated the strongest association with smash accuracy, followed by shoulder flexibility and wrist flexibility. These findings suggest that upper-extremity flexibility and strength are important physical factors associated with badminton smash performance. Assessment and targeted training of these variables may help coaches and sports physiotherapists optimize smash accuracy and enhance overall performance in sub-elite badminton players. Further studies with larger sample sizes and longitudinal designs are recommended to confirm these findings and establish causal relationships.
